# Supplementary material for: Identifying topology of distribution substation in power Internet of Things using dynamic voltage load fluctuation flow analysis
Source: PeerJ Comput Sci. 2024 Feb 8;10:e1688. doi: 10.7717/peerj-cs.1688 (PMC10909208; doi:10.7717/peerj-cs.1688)
Supplement: Supplementary file 1 [file peerj-cs-10-1688-s003.zip › data pack/T1.odt]

Table 1 Comparison of the running time of different algorithms

| Algorithms | Time consumption /ms | Accuracy (0.9) | Accuracy (0.95) |
| --- | --- | --- | --- |
| Full day data analysis | 16.1 | No | No |
| Sliding time window analysis | 1743.2 | Yes | Yes |
| The proposed improved HMM algorithm | 35.1 | Yes | No |
